# Draft Genome Sequence of Antarctic *Pseudomonas* sp. Strain KG01 with Full Potential for Biotechnological Applications

**DOI:** 10.1128/genomeA.00906-15

**Published:** 2015-08-20

**Authors:** María S. Pavlov, Felipe Lira, José L. Martínez, Jorge Olivares, Sergio H. Marshall

**Affiliations:** a Laboratorio de Genética e Inmunología Molecular, Instituto de Biología, Facultad de Ciencias, Pontificia Universidad Católica de Valparaíso, Valparaíso, Chile; b Departamento de Biotecnología Microbiana, Centro Nacional de Biotecnología CSIC, Madrid, Spain; c Fraunhofer Chile Research Foundation, Center For Systems Biotechnology, Santiago, Chile

## Abstract

We report here the draft genome sequence of a free-living psychrotolerant, *Pseudomonas* sp. strain KG01, isolated from an Antarctic soil sample and displaying interesting antimicrobial and surfactant activities. The sequence is 6.3 Mb long and includes 5,648 predicted-coding sequences.

## GENOME ANNOUNCEMENT

The genus *Pseudomonas*, described as early as 1894, is at present considered one of the most diverse and ubiquitous bacterial genera whose species have been isolated worldwide in all kinds of environments, from the poles to the tropics (1). The genus comprises a large number of strains able to produce a complex array of secondary metabolites with biotechnological relevance (2, 3). The recent technical advances in the genomic and metabolomic fields combined with sequencing of Antarctic microorganisms constitute a pivotal tool to augment traditional methods of studying and discovering valuable natural products (4). This *in silico* approach has successfully been demonstrated in the discovery of new antibiotics and plant growth-promoting substances produced by several *Pseudomonas* spp. isolated from temperate-latitude environments (5).

In this framework, we present here the draft sequence of the genome of *Pseudomonas* sp. strain KG01, a strain isolated from Antarctic soil with a full diversity of relevant bioactivities, such as surfactant production and antimicrobial compound secretion (M. S. Pavlov, unpublished data).

To access the genomic information, DNA was extracted using the Gnome DNA isolation kit (MPI Biomedicals, CA) from an overnight bacterial culture and subjected to whole-genome shotgun sequencing. Paired-end libraries, with an average insert size of 300 to 400 bp, were generated using a read length of 2 × 300 bp. Sequencing was performed using Illumina technology, according to the manufacturer’s protocols for Illumina MiSeq at the facilities of Parque Científico de Madrid (Madrid, Spain). Shotgun sequencing produced a total of 2.054.712 × 2 reads. Raw sequences were filtered, and high-quality reads were assembled *de novo* using the MIRA 4.0 software (6) with the parameters established for the correct orientation and insert size determination. The resulting contigs were manually reassembled using Gap5 (Staden package version 2.0.0 b10) (7) to increase their length. The final KG01 assembly included 48 contigs (88.8-fold coverage), with the largest contig measuring 0.70 Mb. The draft genome sequence of *Pseudomonas* sp. strain KG01 comprises a total consensus sequence with an estimated size of 6.3 Mb and 60.1% G+C content.

Gene prediction and annotation were performed using the RAST server (http://rast.nmpdr.org/) (8). Using this approach, 5,648 coding sequences and 59 tRNA genes distributed in 16 scaffolds in the genome of *Pseudomonas* sp. strain KG01 were predicted.

A pyocin-like prophage sequence integrated between *mutS* and *recA*-*recX* (contig 1), two previously described siderophore biosynthetic gene clusters for *Pseudomonas* (pyoverdine, contig 2; pseudomonine, contig 161), an aryl-polyene gene cluster (contig 7), and an ectoine production gene cluster (contig 9) were identified using the antiSMASH server. In addition to previously *in vitro*-acquired evidences, the actual genomic information suggests a putative high potential of *Pseudomonas* sp. strain KG01 to produce biotechnological valuable substances with applications in different fields of biotechnology.

### Nucleotide sequence accession numbers.

This whole-genome shotgun project has been deposited at DDBJ/EMBL/GenBank under the accession no. LFMW00000000. The version described in this paper is version LFMW01000000.
